# Diseases of Children—Palatable Drugs for Children

**Published:** 1883-12

**Authors:** Frederick Churchill

**Affiliations:** Surgeon to, and Member of the Drug Commtttee, Victoria Hospital for Children


DISEASES OF CHILDREN—PALATABLE DRUGS FOR CHILDREN. FREDERICK CHURCHILL, M.D., F.R.C.S., Surgeon to, and Member of the Drug Commtttee, Victoria Hospital for Children, contributes the following to the *British Medical Journal:* '

We owe it, probably, much to the persistency with which practitioners of the sterner sort have impressed their rhubard and black draughts upon rebellious, in defiance of the protestations of nurses and mothers, that "the tasteless globule" has found such favor with the weaker sex. I could tell of several cases where the children have been entrusted to the care of a homœopath, while the parents luxuriate under the usual heroic treatment of the orthodox practitioner. We must not forget to swim with the tide. Children of this enlightened age are far more pampered and spoilt than those of the previous generation. Mothers seem unable to control their feelings; or, it may be, that, with a smattering of physiclore, they find that there is no longer any necessity to cling to the once inevitable and nauseous potion. We must say a word, too, for the children. None of us like compulsion. It must not be forgotten that there is often more harm done to a child's nervous system, by cramming the draught down its throat, than all the good the nauseous drug was supposed to effect. Children will often take days to recover their equilibrium after a protrected encounter with the medicine-glass in the nursery, under the stern discipline of a would-be conscientious nurse. Judging from the varied susceptibilities and dispositions of the children under my care, some of them having very resolute wills, others possessing—I cannot say endowed with—mothers of a pronounced æsthetic temperament, to whom everything is a bore, except a novel to read, and a sofa to lie upon, it becomes most important to formulate a line of treatment that will satisfy such requirements.

This class of children are generally ruled by a domineering old woman they call "nurse," displaying a maximum of "tall talk," with a minimum of what she delights to call "common sense" (and very common, indeed, it proves to be). The medical man must cultivate a habit of attacking such a stronghold of prejudice and conceit by a series of carefully planned flank movements, in such a way that the nursery magnate may be drawn, against her own convictions, into a pliable frame of mind, sufficient to enable the medical man's physic and regimen to stand a chance of being attended to.

To attempt to invade the sanctum of a nursery where the lady-paramount is cajoled into the idea that "nurse is a treasure," and prefers rather to foster the notion, than to care to have her eyes opened to the actual state of reigning ignorance, requires all the practical art of the medical man gradually to overcome and remedy.

Undoubtedly the ailments under which children for the most part suffer, belong to the preventable class. They are due sometimes to overfeeding; very often to neglect, especially of the calls of nature; and very much to general bad management. With this view, it may be well to presume that the best and most approved mode of treatment for habitual torpidity of the bowels is not medicine, but an enema of soap and water, with occasionally a little castor or olive oil added to the injection. If this do not succeed, and the child's appetite begins to fail, it is an indication for administering medicine by the mouth.

Fortunately, the art of the apothecary comes in to our aid, and we are now enabled to give the most nauseous of drugs—castor-oil—absolutely free from taste and smell, while it retains the full aperient properties of ordinary castor-oil. Messrs. Allen and Hanburys themselves advise that it should be shaken up with three or four times its bulk of hot milk. The viscidity of the oil is thus avoided, and the emulsion produced is scarcely distinguishable from warm rich milk.

If it be desirable to administer an aperient that will act more directly on the liver, and to avoid the unpleasant effects which often arise after taking "oil," the compound rhubarb pill will be found a servicable aperient. Of course, some new method for its administration will be desired, which I shall now detail. Either an ordinary five-grain pill may be cut up, and a portion of it broken in small pieces may be buried in a chocolate cream, which the youngest child will take with avidity; or for children of say five years and upward, I have given one-haf and onefourth of a grain of this pill, thinly coated. Half a dozen or so may be taken, like "hundreds and thousands," and washed down with milk or water.

The medicated fruit lozenges are very useful, *e.g.*, tamar indien and laxora lozenges. Podophyllin is probably one of the active ingredients in these lozenges. Only a small portion of a lozenge must be given to a child. The objection found with these is that they sometimes "gripe" the little patient. Next to these, perhaps, in efficiency and palatability, is the compound licorice powder, containing senna powder. About a teaspoonful stirred up with warm milk may be taken at bedtime, and a little chloric ether added (about ten to twenty drops). Very few children will object to take fluid magnesia or the calcined magnesia, especially if flavored with the flavor of mulberry or orange.

I have succeeded in making the taste of many powders by the addition of powdered "rose" lozenges. I very seldom prescribe Gregory's powder on account of its nauseous character and bulk. I prefer to combine the rhubarb with bicarbonate of soda about five grains of each. This makes a much more miscible and manageable powder. Given in jam, honey or golden syrup; the taste is altogether covered.

Children will sometimes take the "baum de vie," or decoction of aloes, without objecting much. A little of this rubbed into the stomach of infants will suffice sometimes to procure an action of the bowels. The extract of liquorice may be added to the decoction until the bitter taste is sufficiently masked. Children have not really such an aversion to it, for I have known them to lick off the aloes from their fingers when put on to prevent them from sucking them. Powdered aloes, about half a teaspoonful, may be given mixed with brown sugar. The electuary of senna is taken without difficulty by some children, also the syrup of senna and the infusion with prunes. The effervescing purgative lemonade is a very agreeable drink, as also half a seidlitz-powder flavored with lemon-juice.

Turning now to febrifuge mixtures, there is not much need of flavoring to mask the flavor of these. Sweet niter, acetate of ammonia, spirits of chloroform, are all pleasant drugs to take. The nitrate and chlorate of potash are rather saltish, but the sal prunelle and Wyeth's compressed tablets will be taken by the bigger children without much protest. The syrups of orange, lemon and mulberry will come in as agreeable and cooling adjuncts. Cough-mixtures can generally be made very pleasant by the addition of syrup of squills, of tolu, etc.

As regards tonics, some considerable skill will be necessary efficiently to cover the bitter flavor. Children will take the saccharated carbonate of iron very well, and also steel wine; but if we attempt to give the bitter infusions, there is sure to be rebellion in the nursery. Quinine—one of the most valuable medicines for children—can be given without difficulty, either in the form of pill, or, which I prefer, dissolved in syrup of orange, without the addition of any water. This effectually covers the flavor. Quinine wine is useful for the elder children.

Chemical food is, of course, taken with relish, and if recently made is a serviceable tonic; but the phosphates, from their insolubility, throw down very much. The compound solution of the hypophosphites, in ten-minim doses, and the hypophosphite wine, forms a perfect substitute for Parrish's food. Besides having iron in a form which is easily absorbed, the hypophosphite of magnesia serves as a useful antacid and stomachic in this combination.

With a view of putting into practical form these two suggestions, and to systematize the irregular but constant attempts of mothers to keep a little dispensary of their own, I have instructed Messrs Savoy and Moore to fit up a nursery medicine-chest, with a companion guide, to assist mothers, especially those residing in the colonies and far away from medical aid, to treat their own children in such emergencies.

---

Dr. Frank O. Stockton has been elected to the professorship of Diseases of the Nose and Throat in the College of Physicians and Surgeons of Chicago.

